# MiRBooking simulates the stoichiometric mode of action of microRNAs

**DOI:** 10.1093/nar/gkv619

**Published:** 2015-06-18

**Authors:** Nathanaël Weill, Véronique Lisi, Nicolas Scott, Paul Dallaire, Julie Pelloux, François Major

**Affiliations:** Institute for Research in Immunology and Cancer, and Department of Computer Science and Operations Research, Université de Montréal, PO Box 6128, Downtown Station, Montréal, Québec H3C 3J7, Canada

## Abstract

In eucaryotes, gene expression is regulated by microRNAs (miRNAs) which bind to messenger RNAs (mRNAs) and interfere with their translation into proteins, either by promoting their degradation or inducing their repression. We study the effect of miRNA interference on each gene using experimental methods, such as microarrays and RNA-seq at the mRNA level, or luciferase reporter assays and variations of SILAC at the protein level. Alternatively, computational predictions would provide clear benefits. However, no algorithm toward this task has ever been proposed. Here, we introduce a new algorithm to predict genome-wide expression data from initial transcriptome abundance. The algorithm simulates the miRNA and mRNA hybridization competition that occurs in given cellular conditions, and derives the whole set of miRNA::mRNA interactions at equilibrium (microtargetome). Interestingly, solving the competition improves the accuracy of miRNA target predictions. Furthermore, this model implements a previously reported and fundamental property of the microtargetome: the binding between a miRNA and a mRNA depends on their sequence complementarity, but also on the abundance of all RNAs expressed in the cell, i.e. the stoichiometry of all the miRNA sites and all the miRNAs given their respective abundance. This model generalizes the miRNA-induced synchronistic silencing previously observed, and described as sponges and competitive endogenous RNAs.

## INTRODUCTION

MicroRNA (miRNA) genes are transcribed into primary transcripts, which are processed into mature single-stranded RNA molecules of ∼22 nucleotides (1). Mature miRNAs are incorporated in the RNA-induced silencing complex (RISC), which initially exposes seed nucleotides 2–5 to the surface that serve as an hybridization template with a complementary site on a messenger RNA (mRNA) (2–4). Base pairing of these positions further expose subsequent seed nucleotides for base pairing (5). When a mRNA is bound by one or numerous miRNAs, it is subject to silencing, either by translational repression, or by degradation, the latter case being dominant (6). Determining the silencing effect exerted on each gene is key to studying cell behavior. This is currently accomplished using various experimental techniques, such as luciferase reporter assays (2,7–8), microarrays (9,10) and RNA-seq (11), variants of SILAC (stable isotope labeling by amino acids in cell culture) (12,13), and variants of CLIP (crosslinking immunoprecipitation) (14–16). Results from these techniques showed that: (i) each miRNA can target several mRNAs; (ii) each mRNA can be the target of several miRNAs and (iii) most mRNAs are subject to miRNA-induced silencing. Accumulating evidences also indicate that (iv) miRNA-induced silencing is subject to cellular conditions (17–20).

Determining the silencing effect applied to each gene using experimental methods is costly, and cannot currently be applied to all desired cell conditions. For instance, experimentally measuring the target and off-target effects of hundreds of small interfering RNAs and in hundreds of cellular conditions is elusive. Alternatively, computational methods are not hindered by such concerns, given an algorithm could predict accurately and in satisfactory runtimes the degrees of silencing induced on each gene. However, none designed for this task has ever been proposed.

Here, we introduce miRBooking, a one-of-a-kind algorithm to address this task computationally. MiRBooking infers the whole set of miRNA::mRNA interactions by simulating the RNA competition to hybridize, finding the stable state at equilibrium (microtargetome), and estimating the miRNA-induced silencing (miS) levels applied to each gene. To simulate competition between miRNAs and mRNAs, we considered the stable marriage problem (SMP) (21). Gale and Shapley defined the formation of stable marriages as an abstract idea in the 60's. It was illustrated by men competing for and proposing women for marriages to establish stable couples by respecting their individual preferences.

By analogy, miRNAs and mRNAs form couples. The preferences are defined by a combination of their absolute abundance obtained from microarray or RNA-seq data, and binding energies expressed as hybridization probabilities (HP). The HPs are based on sequence complementarity between miRNA seeds and miRNA recognition elements (MREs). MirBooking is in-context, provides extended target site information, only requires expression data of the initial cellular conditions, finds microtargetomes that are in agreement with those determined by CLIP experiments, and improves on the accuracy of any other method to predict effective miRNA targets. Finally, miRBooking was validated on two different genome-wide experimental datasets.

## MATERIALS AND METHODS

### Stable marriage algorithm

At the beginning, each man and woman has a list of preferences. Each man proposes the next woman in his list. If the woman is free, she accepts. If she is already married to another man, then, if she prefers the man with whom she is currently married, then she rejects the new proposal, or otherwise the marriage is broken and she rematches with the new man. A man who is rejected proposes the next woman in his list. The algorithm stops when no more stable marriages can be formed, i.e. when no more marriages can favor both party. If the preference lists include all members of the other gender and are ordered from best to worst, then the resulting set of couples is both stable and unique (22).

### RNA abundance of cell lines

#### mRNAs

Using known absolute quantifications of eight mRNAs in human CD4+ and CD8+ cells (23), we applied a linear regression of *log* transformed data (*R*^2^ = 0.5) to convert sets of relative gene expression data into absolute quantities. These absolute expression data were used as a reference to convert any other gene expression data into absolute quantities by fitting the mean and the standard deviation of the *log* transformed data of the considered gene expression to the reference.

#### miRNAs

Absolute quantifications of miRNA expression data were determined from liver samples (24). It was observed that the median and the maximum quantities of miRNAs are respectively 633 and 52 567 copies per cell. These values were used to transform microarray expression data into absolute quantities using a linear regression of the *log* transformed data to adopt the same median and maximum.

#### Datasets

We used the GSE5949 and GSE26375 samples from the Gene Expression Omibus (GEO) (25) to quantify respectively the mRNAs and miRNAs in 41 different cell lines. For each cell line, cDNA hybridization was performed on Affymetrix Human Genome U95 Array, a combination of five microarrays. To avoid a bias among the five microarrays, we realigned the expression data using the *log* transformed expression value of two control genes, *GAPDH* and *ACTB*. For the probes matching more than one mRNA, we considered the longest 3′ UTR of this gene as defined by the RefSeq annotation. The result of this procedure is a set of 41 cell lines in the format of mRNA and miRNA absolute quantities. With this approach, we obtained a good correlation between the absolute quantifications of the DU145 cell line from microarrays versus RNA-seq (*R* = 0.56).

### Seed::MRE hybridization probabilities

The mature miRNA sequences were obtained from miRBase release 20 (June 2013) (26), which contains 1872 human precursor and 2578 mature miRNAs. The mRNA sequences were obtained from NCBI RefSeq release 49 (27), which contains 31 830 human mRNAs. We defined the seed sequence as nucleotides 2–8 of the mature miRNAs (28). We computed the free energies of the duplexes seed::MRE (MicroRNA Recognition Element), Δ*G*, using the MC-Fold software (29), which incorporates the energy contributions of non-canonical base pairs that can possibly form at mismatch positions. The energies of the duplexes were normalized assuming a Boltzmann distribution, and converted into a hybridization probability, HP:

\begin{equation*} {\rm HP}({\rm seed}::{\rm MRE}) = \frac{{e\frac{{ - \Delta G({\rm seed}::{\rm MRE})}}{{kT}}}}{{\sum\nolimits_{h \in {\rm heptamers}} {e\frac{{ - \Delta G({\rm seed}::h)}}{{kT}}} }}, \end{equation*}

where the sum is done over all 4^7^ heptamers, *k* is the Boltzmann constant, and *T* the temperature of the system (to simplify, here we fixed *kT* = 1).

### MiRBooking algorithm

The Gale-Shapley (stable marriage) algorithm consists of iterations of miRNAs proposing MREs until a stable state is reached. Given a cell's conditions, we first established the list of MREs, i.e. the mRNA target sites for which at least one miRNA seed is complementary (maximum of two mismatches), sorted by the absolute abundance of their corresponding mRNA in these conditions. For each MRE, we established the list of its complementary miRNA seeds sorted by their abundance in these conditions, and then sorted by hybridization probabilities of the seed::MRE duplexes. At the beginning of the algorithm, all MREs are free. MiRNA seeds following the sorted order then propose each MRE. The free MREs are assigned a number of miRNA copies equals to:

\begin{equation*} \begin{array}{*{20}l} {Q({\rm seed},{\rm MRE}) = } \\ {\min \left( {q({\rm MRE}) \times {\rm log}_ \propto (q({\rm seed})) \times {\rm HP}({\rm seed}::{\rm MRE}),q({\rm seed})} \right),} \\ \end{array} \end{equation*}

where *q*(MRE) is the initial absolute abundance of the MRE's corresponding mRNA in conditions, *q*(seed) is the current absolute abundance of the miRNA (i.e. at each assignment we subtract *Q*(seed,MRE) from the current abundance of the miRNA), and HP is the hybridization probability of the seed::MRE duplex. Sorting is purely an algorithmic trick to speedup the algorithm. It is possible because the preferences are mutually equivalent, HP(seed:MRE) = HP(MRE::seed), and it makes the algorithm runs faster than by forming and breaking matches. The log of miRNA abundance was prototyped after the observed miRNA dilution by target abundance in a Michaelis–Menten kinetics model (18). The region considered occupied when a miRNA is matched to a MRE has a length of 46 nucleotides centered on the miRNA, as this has been reported to be the minimum footprint span of the RISC (14).

The algorithm iterates until no more seed::MRE pair can be made. The final solution is stable because there is no alternative seed::MRE pair in which both the miRNA and MRE can be individually better off than they would be with their assigned partner, in terms of their relative abundance, hybridization probabilities, and dilution. The algorithm is deterministic and returns a single solution for each input conditions.

### Computing miRNA-induced silencing on each gene

We assume the miRNA-induced silencing on a given mRNA to be proportional to the sum of the contributions of all miRNA copies occupying the multiple copies of this mRNA, and we compute it as the sum of each HP multiplied by a MRE location factor, W:

\begin{equation*} {\rm miS}_C (m_n ) = \sum\nolimits_{{\rm x} \in {\rm seeds};{\rm y} \in {\rm MREs}} {{\rm HP}(x::y) \times {\rm W}(y)}, \end{equation*}

where *C* is the cellular context; *m* is the mRNA expressed at *n* copies in *C; x* is the seed and *y* the MRE; HP(*x*::*y*) is the hybridization probability of forming the duplex *x*::*y*; and, W(*y*) is a contribution factor due to the location of *y* in *m*: W(*y*) = 0.1 if *y* is in the 5′ UTR or coding region (CDS), and W(*y*) = 1.0 if *y* is in the 3′ UTR, as this model is in agreement with experimental observations (30). HP(*x*::*y*) is calculated from the free energy of folding, Δ*G*, of the duplex *x*::*y*.

### Optimization

MiRBooking has two parameters, *τ* and *α. τ* is a threshold on the HP, so that HP(seed::MRE) ≥ *τ*. A large value of *τ* results in miRBooking matching miRNAs and MREs with higher complementarity. *α* is the base of a logarithm, which dilutes the number of miRNA copies assigned to a mRNA when they match during the running of the stable marriages algorithm. A large value of α distributes smaller numbers of miRNA copies to more MREs (larger dilution).

We executed miRBooking to obtain the miRNA::mRNA interaction networks in two different cellular conditions: DU145 and PC3. Then, we overexpressed miR-1 and miR-133a by changing their abundance to 100 000 copies, and executed miRBooking again to obtain the modified networks. Then, we compared the computed miS and measured mRNA expression determined by microarray data (Gene Expression Omnibus GSE26032) (31). The microarray sample contained the log_2_ ratio of the expressed mRNA intensities before and after the miRNA overexpressions. Since the platform used to derive the RNA abundance of our cell lines differs from that used for these microarrays, we only considered the genes that were mapped in both. To reduce the impact of the noise, we fixed the log_2_ fold-change thresholds between −0.2 and 0.2. We defined the genes affected by the miRNA overexpression those with a log_2_ fold-change smaller than −0.2. We obtained 9098 and 8582 data points, respectively, when combining the four experiments. We ran miRBooking using series of combinations of *τ* and *α*: *τ* ∈ [0.0050, 0.0055 … 0.2000], *α* ∈ [2, 4, … and 2^24^]. The predictions’ accuracy was evaluated using the Matthews Correlation Coefficient (MCC), and the optimal was reached with *τ* ∈ [0.0175 … 0.0185] and *α* = 512. We used an additional sampling over values of *t* by increments of 0.00001 to optimize further in the optimal range determined in the first sampling to determine that the optimal values were in the interval [0.0179 … 0.0185], and we chose *τ* = 0.0179, the minimum value in this range.

### Validation and comparison

#### pSILAC

We evaluated and compared the accuracy of the miRBooking algorithm with that of the most popular miRNA target prediction programs. A comparison of seven methods (and some of their variations) was made in 2009 (32). Similarly, we compared the most recent versions of four of these previously benchmarked methods. We used the external dataset and benchmarks based on pSILAC data (13,32). This dataset is composed of five miRNA overexpression experiments in Hela cells (let-7b, miR-155, miR-16, miR-1 and miR-30). The protein fold change values induced by these miRNA overexpressions were determined experimentally using the pSILAC method at 8 and 32 h after overexpression (13).

#### Luciferase

Second, we compared the programs using a data set derived from a luciferase screening assay, where expression levels of a luciferase reporter gene with the 3′ UTR of *P21* were measured before and after the overexpression of 266 different miRNAs in Hela cells (7). This screen allowed us to evaluate the predictive power of the programs in overexpression of the miRNAs and reporter gene. The data set is composed of 128 positives and 138 negatives based on observed luciferase fold-change (<1 and ≥1, respectively). For the miRBooking virtual experiments, we used the *P21* 3′ UTR reporter flanked by the Renillia luciferase sequence as described (luc-P21 3′ UTR; Supplementary Figure S1) (7). The abundance of the overexpressed miRNAs was set to 100K copies, whereas the luc-P21 3′ UTR reporter was set to 100 copies. The effect of each overexpressed miRNA on the luc-P21–3′ UTR were measured in individual virtual experiments performed in HeLa cell conditions. We computed and compared the silencing values in normal HeLa cell and miRNA overexpression conditions, and considered positives (downregulation) the miRNAs for which miS_HeLa_(luc-P21-‘UTR_100_) < miS_HeLa+miRNA↑100K_(luc-P21-‘UTR_100_), and negatives the miRNAs for which miS_HeLa_(luc-P21-‘UTR_100_) ≥ miS_HeLa+miRNA↑100K_(luc-P21-‘UTR_100_). For the other evaluated programs, we considered positives the miRNAs predicted to downregulate CDKN1A (RefSeq ID NM_000389).

### Precision, sensitivity and AU-ROC curves

To answer what programs identify the best the highly affected genes after changing the conditions, we used the Precision and Recall model, as well as the Area Under the Receiver Operating Characteristic curve (AU-ROC). The accuracy of a predictive method can be evaluated as a combination of precision and recall measures, and is based on the prediction of the genes found downregulated after the overexpression of various miRNAs. The precision is given by the ratio TP/(TP + FP), and the recall by TP/(TP + FN), where TP is for True Positives; FP, False Positives; and, FN, False Negatives.

For the AU-ROC curves, we defined that a given gene is highly affected if its fold-change is below some threshold, *t*, log_2_(fold-change) < *t*. We evaluated three *t* values: ≥1.0, −0.7 and −0.5. The targets were ordered using the scores returned by each method. The p-values were provided by the R verification package, and computed using the Wilcoxon test. We wanted to test all miRNA target prediction approaches. However, none based on Gene expression correlation or seed-expression hybrid method provided enough predictions to be evaluated in the genome-wide context of the pSILAC dataset composed of 15 734 miRNA::target pairs: 86 positive pairs when *t* = −1.0; 257 when *t* = −0.7 and 514 when *t* = −0.5.

We tested Lasso (33), but it required an appropriate training on HELA cell expression data that are not available or straightforward to generate. The inclusion of an inappropriate training would lead to misinterpretation of the results. We tried the three different datasets provided by the authors, but none allowed us to make predictions on more than 20 genes. We experienced a similar story with the hybrid method HOCTAR (34). We thus decided to investigate with two different sequence-matching methods with and without their respective sequence conservation and site accessibility features. We evaluated miRbooking by ordering gene expression fold-change.

### Synchronous silencing

For each mRNA (leader) in DU145 cells (7416 genes), we systematically assigned 100 to 6000 copies by increments of 100, and computed the miS on all other mRNAs (followers). Then, we computed the correlation between the silencing applied to the followers and the abundance of the leaders. This resulted in a non-symmetrical square correlation matrix of 7416 × 7416 entries. We applied a hierarchical clustering over the leader and follower mRNAs using a Ward linkage. The mRNA cluster analysis was made using high enrichments of GO terms derived from the David Bioinformatics Platform (35).

## RESULTS

### Determining the microtargetome using the stable marriage algorithm

We used expression data from real-time PCR and microarray experiments to derive absolute abundances of 41 cell lines (23,24). We precomputed all possible 7-mer duplexes, corresponding to 2^7^ seeds × 2^7^ MREs, and analyzed the distribution of the numbers of mismatches at different HP (Supplementary Figure S2A). The subset of seed::MRE matches equal or over the optimal HP threshold (see ‘Materials and Methods’ section) are constituted of ∼30% and ∼70% perfect- and 1-mismatch duplexes, respectively. These represent almost all perfect-seed matching duplexes, but only about 10% of the 1-mismatch duplexes. Interestingly, in the microtargetomes of three different cell lines (DU145, PC3 and HCT116) computed by miRBooking, we found the reverse ratio of perfect- and 1-mismatch duplexes (Supplementary Figure S2B), i.e. ∼70% of the matches are perfect. This ratio corroborates those observed in maps derived from HITS-CLIP and PAR-CLIP (14,15).

The miRBooking model implements an important property of the microtargetome: the number and diversity of miRNAs occupying a mRNA are determined by sequence complementarity, but also by stoichiometry, i.e. all the MREs and all the miRNAs expressed. The stable marriage algorithm intrinsically implements the stoichiometry. As a result, given cellular conditions the algorithm assigns greater diversity and amounts of miRNA copies to more abundant mRNAs. Indeed, the balance between transcription and translation rates, as well as miRNA occupancies determine the actual expression variations of associated proteins.

**Figure 1.**
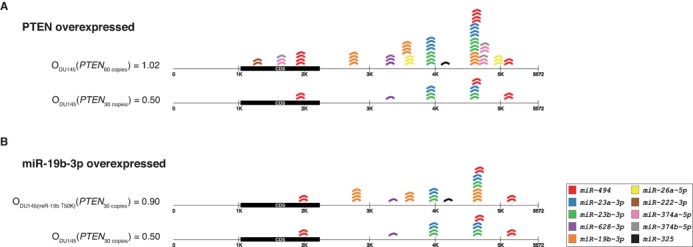
*PTEN* miRNA occupancies vary in different DU145 conditions. *PTEN* is represented by a line; coding region in black (CDS). Colored miRNA copies represent each occupied MREs. (**A**) *PTEN* expressed at 30 copies attracts two copies of miR-494 (red squares; two sites), one copy of miR-628–3p (purple square; one site), two miR-23a-3p (blue squares; two sites), and two copies of miR-23b-3p (green squares; two sites). This leads to occupancy of 0.5; or, 1.02 when *PTEN* is expressed at 60 copies. (**B**) In endogenous levels in DU145 cells miR-19b-3p is expressed at around 4600 copies, which leads to a *PTEN* occupancy of 0.50; or, 0.90 when miR-19b-3p is overexpressed at 50 000 copies.

To exemplify the property, we defined a mRNA occupancy, O, as the average of miRNA copies per mRNA copy. Consider *PTEN*, which is expressed at 30 copies in DU145 cells. MiRBooking assigns to *PTEN* 15 miRNAs (one or more copies of miR-494, miR-222–3p, miR-23a-3p, and miR-23b-3p) (Figure 1A). In these DU145 cells, the occupancy of *PTEN* is 0.50, O_DU145_(*PTEN*_30_) = 15 miRNAs/30 *PTEN* copies = 0.5 miRNA per copy. If *PTEN*'s abundance doubles in these cells, then its occupancy increases to 1.02.

The modification of the abundance of a miRNA can also modify the occupancies. For example, if we increase the abundance of miR-19b in DU145 cells to 50 000 copies, the occupancy of *PTEN* increases to 0.90 (Figure 1B). By comparing miS values before and after the overexpression of miR-19b, we can predict the downregulation of *PTEN* after the overexpression of miR-19b: miS_DU145(miR-19b↑50K)_(*PTEN*_30_) = 1.32 is greater than miS_DU145_(*PTEN*_30_) = 0.59.

Different cellular condition are likely to change the microtargetome. To exemplify this, consider the same abundance of *PTEN* but in HCT116 cells. Both the occupancy and miRNA species on *PTEN* differ when compared to the microtargetome derived from DU145 cells. MiRBooking matches six copies of miR-494, six copies of miR-19b-3p, and one copy of miR-222-3p. MiR-19b and miR-222 were not occupying *PTEN* in DU145 cells, and miR-628-3p and miR-23a/b, which were, disappeared from the microtargetome of HCT116 cells (not shown).

### Validation and comparison of the accuracy of target predictions

The stoichiometric feature of the microtargetome and the miRBooking algorithm can be validated by reproduce experimental data. To make sure that the training set does not bias the validation, we excluded it. To measure if considering stoichiometry and simulating the RNA competition provide any gain in accuracy, we compared miRBooking with other miRNA target prediction methods. We used two independent benchmark datasets: one is based on pSILAC and the other on luciferase reporter data.

**Figure 2.**
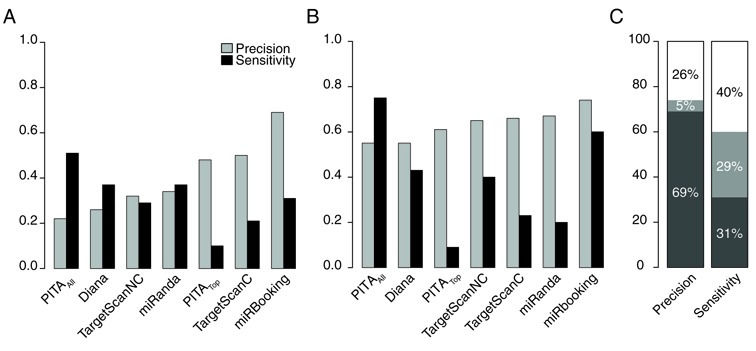
Simulating hybridization improves the accuracy of miRNA target prediction. The predictive power is represented as a combination of precision and sensitivity measures and is based on the prediction of the genes found downregulated after the overexpression of various miRNAs. The modes of some programs are indicated in their names: TargetScan C for the predictions that consider evolutive conservation, or NC for the predictions that do not account for evolutive conservation; PITA_TOP_ for the subset of most confident predictions, or PITA_All_ for all predictions. (**A**) The pSILAC dataset. TP: the genes measured downregulated by pSILAC that are predicted either targeted or downregulated by the programs, independently of the scores. FP: the genes predicted targeted or downregulated by the programs that were measured either non affected or upregulated by pSILAC. FN: the genes that were measured downregulated by pSILAC that are not predicted by the programs. (**B**) The luciferase reporter gene dataset. TP: the miRNA that reduced the luciferase signal and were predicted by the programs to either target or downregulate *P21*, independently of the scores. FP: the miRNA predicted to target or downregulate *P21* by the programs that did not affect or increased the luciferase signal. FN: the miRNA that reduced the luciferase signal, but were not predicted by the programs to target or downregulate *P21*. (**C**) Comparison of the precision and sensitivity obtained by miRBooking in the pSILAC (dark gray) and luciferase (light gray) benchmarks; gap to perfect precision and sensitivity indicated in the white areas.

The accuracy of predictive methods is usually assessed using two measures: precision and recall. The precision is the fraction of retrieved instances that are relevant, while recall (also called sensitivity) is the fraction of relevant instances that are retrieved. In Figure 2, we can see that miRBooking is the only model with a precision over 50% in the pSILAC dataset, whereas it has the highest precision in the luciferase dataset (with a sensitivity of near 60%, which is the second best). The model with the best sensitivity is PITA_ALL_, but at the price of having the worst precision in both datasets. Many alternative approaches to target prediction beyond seed matching were tested using miRBooking without any improvement. These include: considering the A in the mRNA's first position (2); the accessibility of the binding site defined by mRNA folding energies (36); site cooperativity (2); and, the energy of folding of the entire miRNA::MRE (37).

We also evaluated and compared the sequenced-based methods using area under ROC curves (AU-ROC) on the pSILAC dataset. We can see in Supplementary Figure S3 that miRBooking at different thresholds obtain larger area under the curves and *P*-values than any other method. We also observed that miRBooking's AU-ROC curves increased with higher values of the threshold. The other evaluated methods showed the opposite tendency. The sequence matching methods performed better when no additional features (e.g. conservation or accessibility) were included. Noteworthy is the fact that the methods based on gene expression correlation could not be compared, as they did not made enough predictions to provide statistics on the pSILAC dataset.

### Synchronous silencing

**Figure 3.**
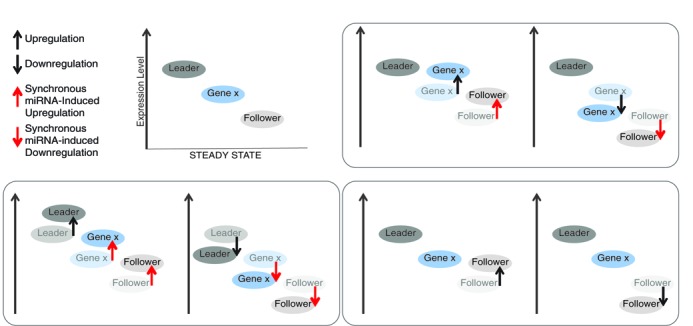
MiRNA-induced silencing synchronizes gene expression. Gene x and the leader and follower genes can share or not the same miRNAs. The up- or down-regulation of a leader entails the regulation of gene x and followers; regulation of gene x entails that of the followers, but not that of the leaders; and, regulation of the followers has no impact on gene x or the leaders.

The stoichiometric feature of the microtargetome logically implies the crosstalk modulation of gene expression, as observed by many either as sponges (38) or competitive endogenous RNAs (19). Essentially, given cellular conditions, increasing the expression of a mRNA sequestered miRNAs (by competition), leaving less to be distributed amongst the others. All such cascading effects simulated by miRBooking are summarized in Figure 3. The silencing level applied to a given mRNA can be affected by changing the expression of another. The leader mRNA is the one that affects the silencing of the other, whereas the follower is the one that gets affected. An interesting attribute arising from this model is that the leaders and followers do not need necessarily to share common miRNAs. The effect can be propagated indirectly through a network of other miRNAs and mRNAs.

**Figure 4.**
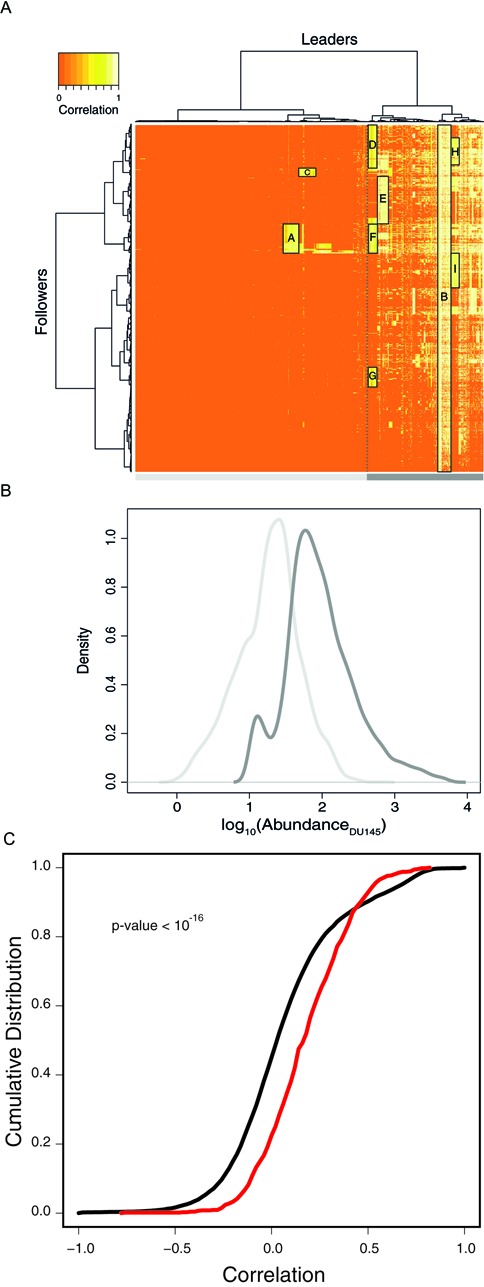
MiRNA-induced silencing and mRNA abundance correlates. (**A**) Correlation matrix between the abundance of leader genes and the miS applied to all others in DU145 cells, from 0 (no correlation, orange) to 1 (perfect correlation, white). Hierarchical clustering separates the leaders in two groups (left and right of the dotted line). Groups of leaders and followers are shown in rectangles labeled A to I. (**B**) Messenger RNA abundance density distribution of the two groups of leaders: lowly expressed genes (pale gray curve) and highly expressed genes (dark gray curve). (**C**) The cumulative distribution of the correlation between the abundance of *PTEN* and that of all genes expressed (black), compared to the cumulative distribution of the correlation between the abundance of *PTEN* and that of its leaders and followers (red), in 41 cell lines and measured using microarrays.

Using miRBooking simulations, we computed the correlations between the abundance of a leader mRNA on the miS it induces on follower mRNAs in five cell lines: DU145 (Figure 4A), and K562, HCT116, MCF7 and BT549 (Supplementary Figure S3). Note that in the current miRBooking model, the miS equation was not adjusted to fit experimental values. Therefore, the goal here was to detect the presence of links between leaders and followers, but not to precisely quantify them. Remarkably, the hierarchical clustering highlights the high expression of most leaders (right side of the heat map with more white color), and the low expression of the followers (left side of the heat map with more orange color). This separation between the highly and lowly expressed groups is made more clear when plotting the expression level distributions of both, as shown in Figure 4B.

The miS of the genes in the groups labeled A to I in the heat map highly correlate synchronistically (Figure 4A). For instance, group B has the highest mean correlation (*R*_mean_ = 0.78). This group contains 292 mRNAs related to the GO-terms ‘translational elongation’ and ‘ribosomal protein’ (*P*-values < 10^−62^ and 10^−49^, respectively). It contains over 20% ribosomal protein genes (RPGs) (63/292). Group A is the largest highly correlated group in the low expression region of the heat map. It is defined by 322 leaders, including 59 transcription factors (*P*-value < 10^−11^) and 622 followers, including 97 transcription factors (*P*-value < 10^−6^). These genes relate to the GO-term ‘regulation of transcription, DNA-dependent’. The leaders and followers of the other groups (C to I) are similarly coupled as those in groups A and B. However, no significant enrichment in GO terms was found for these groups. We found interesting that although the miRNAs involved in the miS applied to each gene vary from one cellular context to another (stoichiometric feature), the synchronistic relationships among the genes is rather stable across the different cell lines (Figure 4A and Supplementary Figure S4).

To appreciate the contribution of leader and follower genes to any particular one, consider the expression of *PTEN* and the comparison of the cumulative distribution of the correlations between its abundance and all other genes expressed in 41 cell lines, with that between *PTEN* and the genes identified as its leaders and followers by miRBooking. Note that the cumulative distributions shown in Figure 4C are based on experimental data alone. These data from 41 cell lines corroborate the synchronous regulation of *PTEN* and its leader and follower genes (*P*-value = 10^−16^), as inferred by miRBooking.

Regulatory synchronicity represents a programmable device that allows cells to link transcription and translation. Ultimately, at the top of the hierarchy, a group of mRNAs sufficiently abundant could synchronize the expression a large fraction of all other mRNAs, e.g. group B in Figure 4. This group is enriched by RPGs which by themselves represent a large fraction of the total mRNA abundance, which has the potential to sponge an important fraction of the total miRNA abundance. The RPG group is thus a serious candidate to global mRNA regulation, which could, for instance, self-establish a balance between the number of transcripts and their translation for cellular homeostasis. In Supplementary Figure S5, we can see that the members of the RPG cluster have shorter sequences than the mRNAs in RefSeq or expressed in DU145 cells. This is somewhat surprising since these global leaders evolved to maximize their competitive effects.

From these results, it would be interesting to investigate the participation of the ribosomal RNAs in cellular homeostasis. Self-regulation between transcription and translation goes back to procaryotes, where these two main regulatory mechanisms are coupled and synchronized in *cis* by operon and riboswitch mechanisms. This necessary synchronization between transcription and translation during the separation of procaryotes and eucaryotes could have evolved through the miRNA-based regulatory mechanism. The synchronized expression of mRNA groups offers in *trans* the same functionalities as those of operons and riboswitches, with malleability and programmable advantages. We thus hypothesize a role of the ribosomal machinery, and in particular of the RPGs, that links its size (abundance) to the numbers of genes that can be expressed and regulated in a given cell.

## DISCUSSION

We showed here that simulating RNA hybridization and accounting for the stoichiometry of the RNAs expressed in given cellular conditions leads to a significant improvement in the accuracy of miRNA target predictions. Interestingly, the sensitivity of the method in a benchmark constituted of a luciferase assay dataset increased considerably compared to that obtained from a pSILAC dataset. The difference between luciferase and pSILAC assays is that the overexpression of a reporter gene in the former escapes the competition. In general, the validation of miRNA::mRNA interactions in luciferase or overexpression experiments represents an artifactual situation that may not reflect the physiological environments. Our model suggests that the low silencing effects measured in such experiments is due to the fact that a highly expressed mRNA is already collectively targeted by many other miRNAs. In physiological conditions, the effect of increasing the abundance of a miRNA may be more important on a lowly expressed gene. The level of silencing applied to any gene highly depends on the cellular context, and taking this feature into account in the design of RNAi-based therapeutics is critical.

Three categories of miRNA prediction software were benchmarked, and only sequence matching methods provided enough predictions to be compared using genome-wide benchmarks. Surprisingly, sequence matching methods provided better results when additional features were not considered. TargetScan gave better results in terms of AU-ROC curves and p-values when the sequence conservation was not used. We observed the same behavior with PITA when the site accessibility parameter was not activated. This indicates that the initial objective of using these features, i.e. to decrease the number of false positives, came with a higher cost, that of increasing the rate of false negatives.

**Figure 5.**
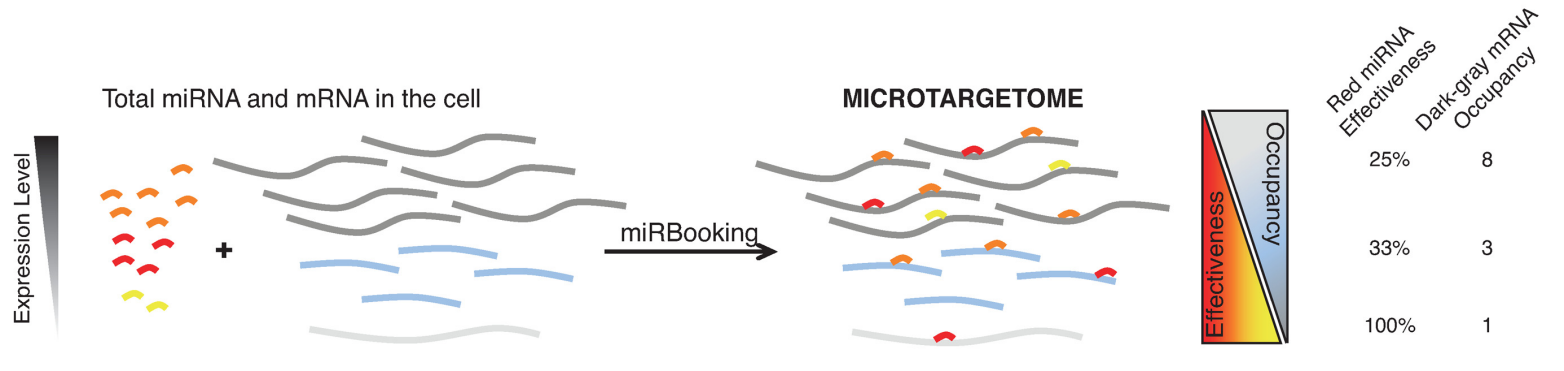
MiRBooking computes the microtargetome from total miRNA and mRNA abundance. From total miRNAs and mRNAs of the cell, the miRBooking algorithm determines the microtargetome. It implements a fundamental property of the microtargetome: as the abundance of a mRNA increases, its occupancy increases (blue triangle), but the effectiveness of any given miRNA decreases (yellow/red triangle).

The miRBooking model uses a stable marriage algorithm that intrinsically implements the stoichiometric feature of the microtargetome, summarized in Figure 5. This model highlights a miRNA-induced synchronistic silencing mechanism that generalizes the concepts previously described in the literature as miRNA sponges or competitive endogenous RNAs. This phenomenon must be accounted for when using RNA interference, as well as knockdown or knockout experiments, as modifying the expression level of even a single gene can have drastic effects on the expression of several others.

## SUPPLEMENTARY DATA

Supplementary Data are available at NAR Online.

SUPPLEMENTARY DATA
