# Supplementary material for: MiRBooking simulates the stoichiometric mode of action of microRNAs
Source: Nucleic Acids Res. 2015 Jun 18;43(14):6730–8. doi: 10.1093/nar/gkv619 (PMC4538818; doi:10.1093/nar/gkv619)
Supplement: SUPPLEMENTARY DATA [file supp_43_14_6730__index.html]

MiRBooking simulates the stoichiometric mode of action of microRNAs — SUPPLEMENTARY DATA 

# MiRBooking simulates the stoichiometric mode of action of microRNAs

## SUPPLEMENTARY DATA

- SUPPLEMENTARY DATA
