# Supplementary material for: MiRBooking simulates the stoichiometric mode of action of microRNAs
Source: Nucleic Acids Res. 2015 Jun 18;43(14):6730–8. doi: 10.1093/nar/gkv619 (PMC4538818; doi:10.1093/nar/gkv619)
Supplement: SUPPLEMENTARY DATA [file supp_gkv619_nar-03296-n-2014-File008.pdf]

AUGGAAGACGCCAAAAACAUAAAGAAAGGCCCGGCGCCAUUCUAUCCGCUGGAAGAUGGAACCGCUGGAGAGCAACUGCAUAAG
GCUAUGAAGAGAUACGCCCUGGUUCCUGGAACAAUUGCUUUUACAGAUGCACAUAUCGAGGUGGACAUCACUUACGCUGAGUAC
UUCGAAAUGUCCGUUCGGUUGGCAGAAGCUAUGAAACGAUAUGGGCUGAAUACAAAUCACAGAAUCGUCGUAUGCAGUGAAAAC
UCUCUUCAAUUCUUUAUGCCGGUGUUGGGCGCGUUAUUUAUCGGAGUUGCAGUUGCGCCCGCGAACGACAUUUAUAAUGAACGU
GAAUUGCUCAACAGUAUGGGCAUUUCGCAGCCUACCGUGGUGUUCGUUUCCAAAAAGGGGUUGCAAAAAAUUUUGAACGUGCAA
AAAAAGCUCCCAAUCAUCCAAAAAAUUAUUAUCAUGGAUUCUAAAACGGAUUACCAGGGAUUUCAGUCGAUGUACACGUUCGUC
ACAUCUCAUCUACCUCCCGGUUUUAAUGAAUACGAUUUUGUGCCAGAGUCCUUCGAUAGGGACAAGACAAUUGCACUGAUCAUG
AACUCCUCUGGAUCUACUGGUCUGCCUAAAGGUGUCGCUCUGCCUCAUAGAACUGCCUGCGUGAGAUUCUCGCAUGCCAGAGAU
CCUAUUUUUGGCAAUCAAAUCAUUCCGGAUACUGCGAUUUUAAGUGUUGUUCCAUUCCAUCACGGUUUUGGAAUGUUUACUACA
CUCGGAUAUUUGAUAUGUGGAUUUCGAGUCGUCUUAAUGUAUAGAUUUGAAGAAGAGCUGUUUCUGAGGAGCCUUCAGGAUUAC
AAGAUUCAAAGUGCGCUGCUGGUGCCAACCCUAUUCUCCUUCUUCGCCAAAAGCACUCUGAUUGACAAAUACGAUUUAUCUAAU
UUACACGAAAUUGCUUCUGGUCCCUAAUCCGCCCACAGGAAGCCUGCAGUCCUGGAAGCGCGAGGGCCUCAAAGGCCCGCUCUA
CAUCUUCUGCCUUAGUCUCAGUUUGUGUGUCUUAAUUAUUAUUUGUGUUUUAAUUUAAACACCUCCUCAUGUACAUACCCUGGC
CGCCCCCUGCCCCCCAGCCUCUGGCAUUAGAAUUAUUUAAACAAAAACUAGGCGGUUGAAUGAGAGGUUCCUAAGAGUGCUGGG
CAUUUUUAUUUUAUGAAAUACUAUUUAAAGCCUCCUCAUCCCGUGUUCUCCUUUUCCUCUCUCCCGGAGGUUGGGUGGGCCGGC
UUCAUGCCAGCUACUUCCUCCUCCCCACUUGUCCGCUGGGUGGUACCCUCUGGAGGGGUGUGGCUCCUUCCCAUCGCUGUCACA
GGCGGUUAUGAAAUUCACCCCCUUUCCUGGACACUCAGACCUGAAUUCUUUUUCAUUUGAGAAGUAAACAGAUGGCACUUUGAA
GGGGCCUCACCGAGUGGGGGCAUCAUCAAAAACUUUGGAGUCCCCUCACCUCCUCUAAGGUUGGGCAGGGUGACCCUGAAGUGA
GCACAGCCUAGGGCUGAGCUGGGGACCUGGUACCCUCCUGGCUCUUGAUACCCCCCUCUGUCUUGUGAAGGCAGGGGGAAGGUG
GGGUCCUGGAGCAGACCACCCCGCCUGCCCUCAUGGCCCCUCUGACCUGCACUGGGGAGCCCGUCUCAGUGUUGAGCCUUUUCC
CUCUUUGGCUCCCCUGUACCUUUUGAGGAGCCCCAGCUACCCUUCUUCUCCAGCUGGGCUCUGCAAUUCCCCUCUGCUGCUGUC
CCUCCCCCUUGUCCUUUCCCUUCAGUACCCUCUCAGCUCCAGGUGGCUCUGAGGUGCCUGUCCCACCCCCACCCCCAGCUCAAU
GGACUGGAAGGGGAAGGGACACACAAGAAGAAGGGCACCCUAGUUCUACCUCAGGCAGCUCAAGCAGCGACCGCCCCCUCCUCU
AGCUGUGGGGGUGAGGGUCCCAUGUGGUGGCACAGGCCCCCUUGAGUGGGGUUAUCUCUGUGUUAGGGGUAUAUGAUGGGGGAG
UAGAUCUUUCUAGGAGGGAGACACUGGCCCCUCAAAUCGUCCAGCGACCUUCCUCAUCCACCCCAUCCCUCCCCAGUUCAUUGC
ACUUUGAUUAGCAGCGGAACAAGGAGUCAGACAUUUUAAGAUGGUGGCAGUAGAGGCUAUGGACAGGGCAUGCCACGUGGGCUC
AUAUGGGGCUGGGAGUAGUUGUCUUUCCUGGCACUAACGUUGAGCCCCUGGAGGCACUGAAGUGCUUAGUGUACUUGGAGUAUU
GGGGUCUGACCCCAAACACCUUCCAGCUCCUGUAACAUACUGGCCUGGACUGUUUUCUCUCGGCUCCCCAUGUGUCCUGGUUCC
CGUUUCUCCACCUAGACUGUAAACCUCUCGAGGGCAGGGACCACACCCUGUACUGUUCUGUGUCUUUCACAGCUCCUCCCACAA
UGCUGAAUAUACAGCAGGUGCUCAAUAAAUGAUUCUUAGUGACUUUACUUGU

Figure S1. Luc-p21-3'UTR Reporter Sequence.

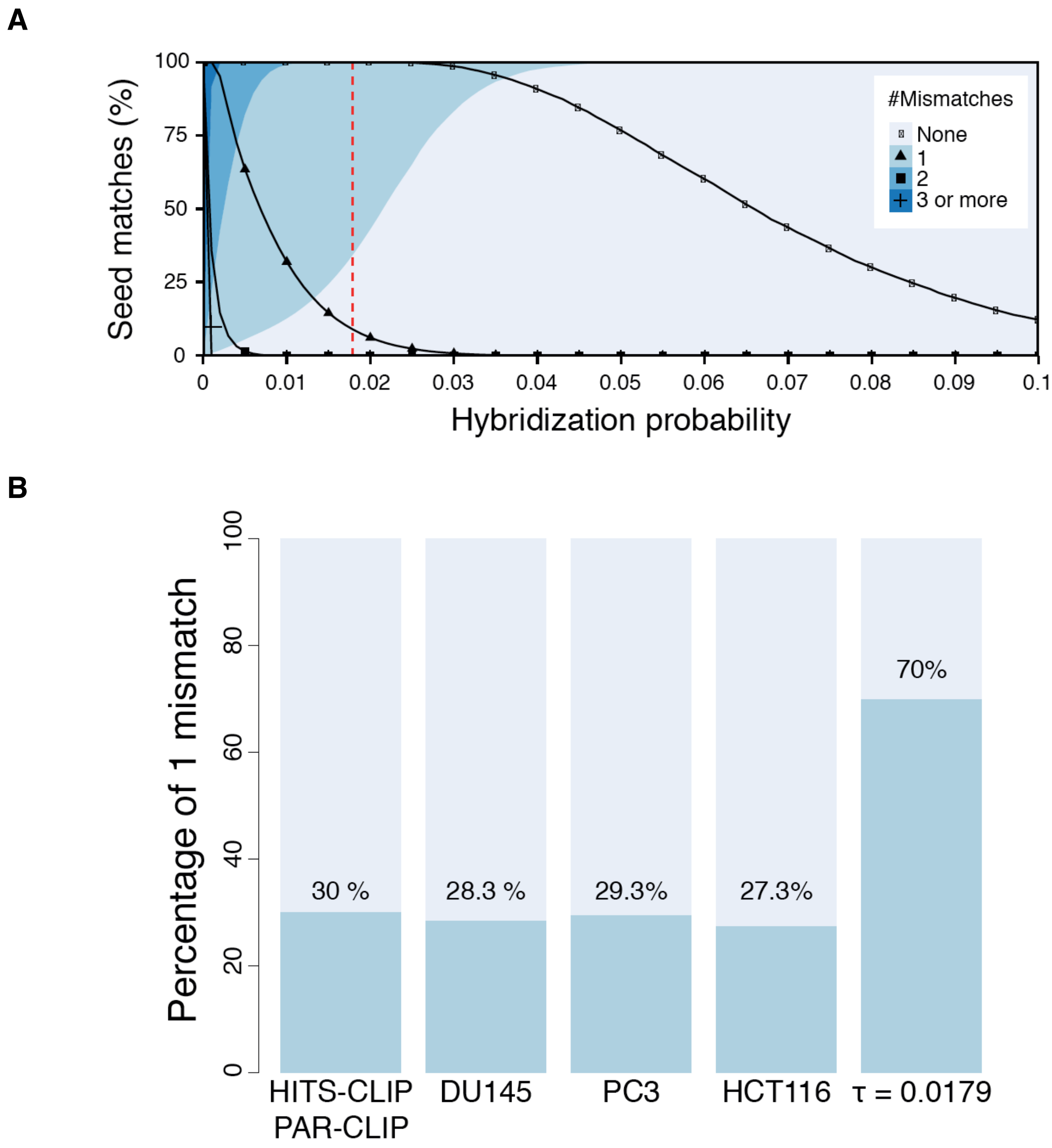

Figure S2. **Hybridization probabilities and proportion of perfect- and imperfect-seed matches**. (A) Proportions of perfect- (None) and imperfect-seed matches (1, 2, 3, or more mismatches) in function of their hybridization probabilities (HP). The optimized HP is shown by a red dotted line ($\tau = 0.0179$; ~30% perfect matches and ~70% 1-mismatches). The curved lines represent the proportions within a group at each HP. At $\tau = 0.0179$, the perfect-match curve crosses the red dotted line near 100%, and the 1-mismatch curve crosses the red dotted line near 10%. (B) The proportion of perfect- (pale gray) and imperfect-seed matches (blue) determined by HITS-CLIP and PAR-CLIP (left), predicted by miRBooking in three cell lines (DU145, PC3, and HCT116), and theoretically possible given the optimized HP, $\tau = 0.0179$.

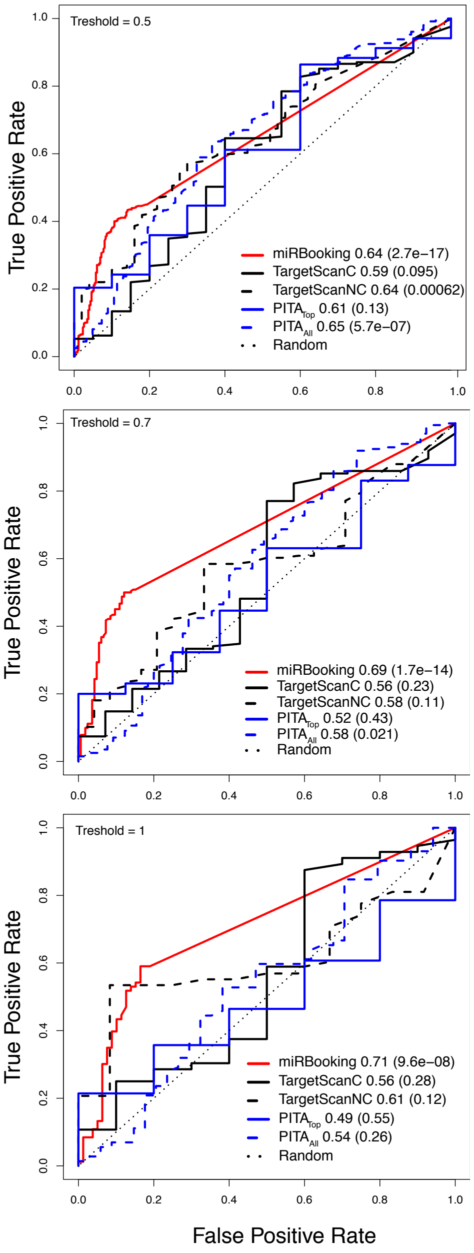

Figure S3. AU-ROC curves at expression fold-change (log) thresholds of -0.5, -0.7, and -1. The evaluated programs are: miRBooking (red), TargetScan (black), TargetScan without conservation (dashed black), PITATop (most confident) predictions (blue), and PITAAll predictions (dashed blue). As a guide, random predictions would generate a AU-ROC of 0.5 (dotted black). The numbers right next to the method names are the area under the curve, and the numbers in parentheses are the p-values associated to the predictions.

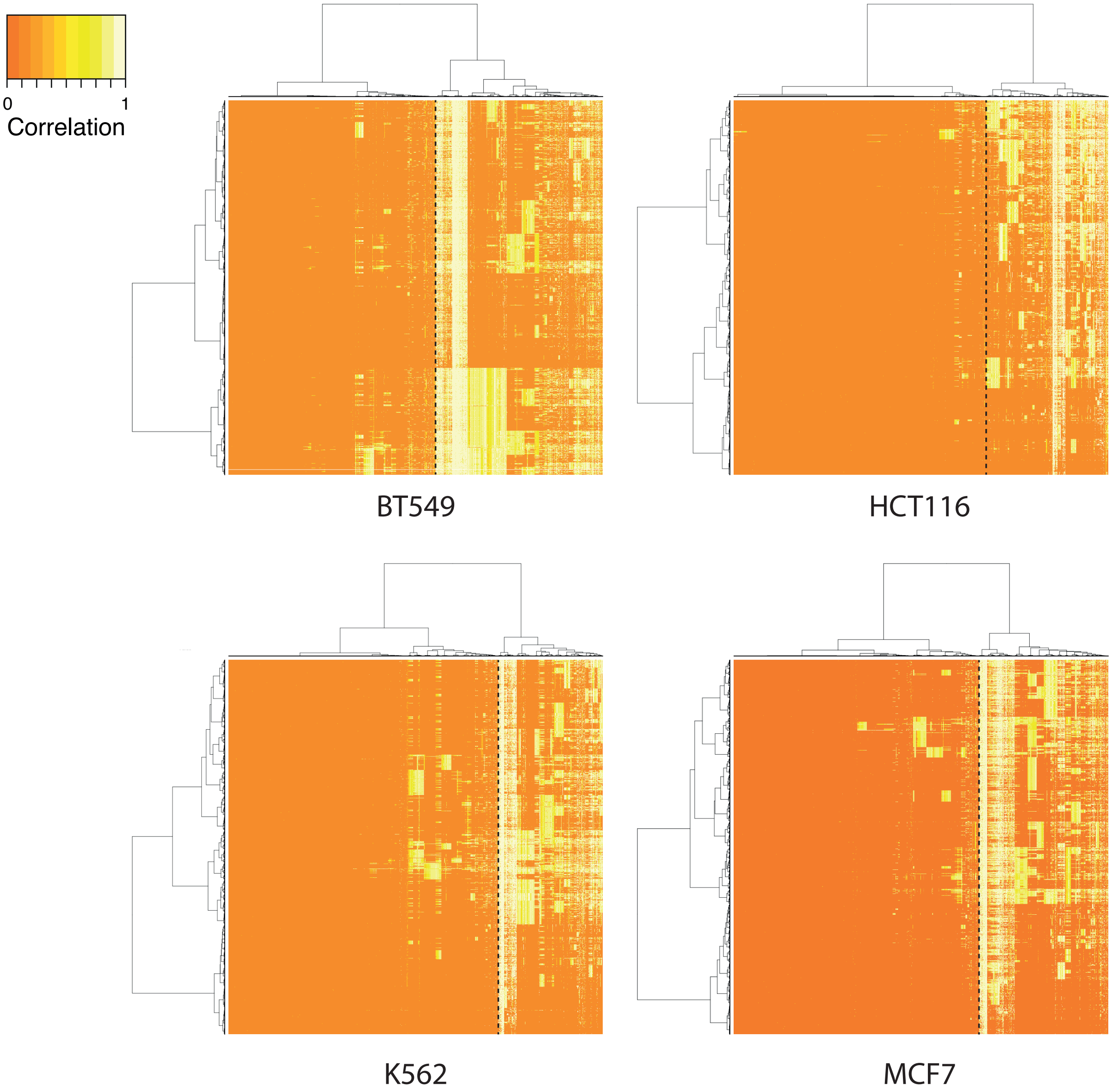

Figure S4. Correlation matrices between mRNA abundance and silencing in four different cell lines. Correlation matrices between the abundance of leader genes and the miS applied on all others in four cell lines, from 0 (no correlation, orange) to 1 (perfect correlation, white). Hierarchical clustering separates the leaders in two groups (left and right of the dotted line). Groups of leaders and followers are shown in white rectangles.

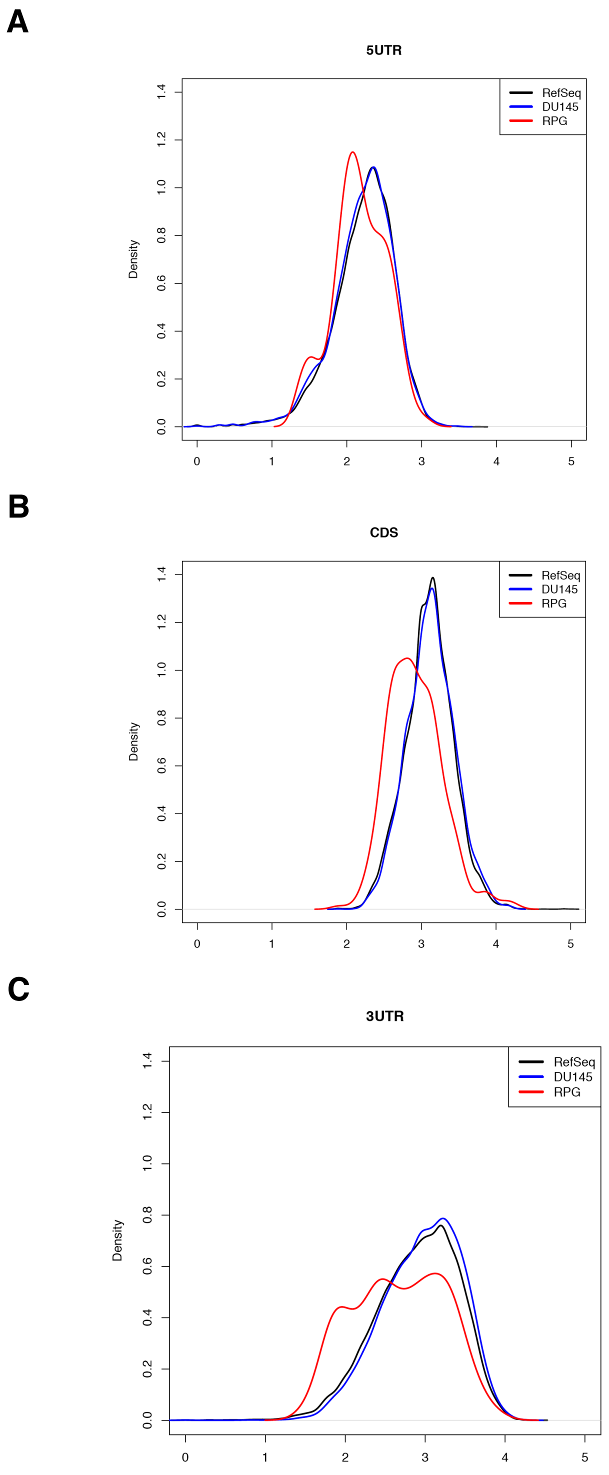

Figure S5. Messenger RNA sequence length distribution. (A) 5'UTRs; (B) CDS; and, (C) 3'UTRs. All sequences included in RefSeq (black), expressed sequences in DU145, and RPG sequences in the cluster B in Figure 4.
